# Pancreas Preservation with a Neutrophil Elastase Inhibitor, Alvelestat, Contributes to Improvement of Porcine Islet Isolation and Transplantation

**DOI:** 10.3390/jcm11154290

**Published:** 2022-07-23

**Authors:** Ryusei Otsuka, Chika Miyagi-Shiohira, Kazuho Kuwae, Kai Nishime, Yoshihito Tamaki, Tasuku Yonaha, Mayuko Sakai-Yonaha, Ikuo Yamasaki, Misaki Shinzato, Issei Saitoh, Masami Watanabe, Hirofumi Noguchi

**Affiliations:** 1 Department of Regenerative Medicine, Graduate School of Medicine, University of the Ryukyus, Okinawa 903-0215, Japan; e184119@eve.u-ryukyu.ac.jp (R.O.); chika@med.u-ryukyu.ac.jp (C.M.-S.); e164157@eve.u-ryukyu.ac.jp (K.K.); e174183@eve.u-ryukyu.ac.jp (K.N.); t97yo.shi328@gmail.com (Y.T.); e174153@eve.u-ryukyu.ac.jp (T.Y.); e164202@eve.u-ryukyu.ac.jp (M.S.-Y.); i.himawari1977@gmail.com (I.Y.); e184120@eve.u-ryukyu.ac.jp (M.S.); 2 Department of Pediatric Dentistry, Asahi University School of Dentistry, Hozumi 501-0296, Japan; isaitoh@dent.asahi-u.ac.jp; 3 Department of Urology, Okayama University Graduate School of Medicine, Dentistry and Pharmaceutical Sciences, Okayama 700-8558, Japan; masami5@md.okayama-u.ac.jp

**Keywords:** pancreas preservation, alvelestat (AZD9668, MPH966), neutrophil elastase (NE), islet transplantation

## Abstract

For pancreatic islet transplantation, pancreas procurement, preservation, and islet isolation destroy cellular and non-cellular components and activate components such as resident neutrophils, which play an important role in the impairment of islet survival. It has been reported that inhibitors of neutrophil elastase (NE), such as sivelestat and α1-antitrypsin, could contribute to improvement of islet isolation and transplantation. In this study, we investigated whether pancreatic preservation with alvelestat, a novel NE inhibitor, improves porcine islet yield and function. Porcine pancreata were preserved with or without 5 μM alvelestat for 18 h, and islet isolation was performed. The islet yields before and after purification were significantly higher in the alvelestat (+) group than in the alvelestat (−) group. After islet transplantation into streptozotocin-induced diabetic mice, blood glucose levels reached the normoglycemic range in 55% and 5% of diabetic mice in the alvelestat (+) and alvelestat (−) groups, respectively. These results suggest that pancreas preservation with alvelestat improves islet yield and graft function and could thus serve as a novel clinical strategy for improving the outcome of islet transplantation.

## 1. Introduction

Pancreatic islet transplantation is a treatment option for patients with type 1 diabetes mellitus [1,2,3,4,5]. Islet transplantation is a minimally invasive approach to β-cell replacement compared with pancreas transplantation. However, the number of islets from one donor pancreas is usually insufficient to achieve insulin independence [1,6,7,8]. The procedure of islet isolation itself destroys cellular and non-cellular components of the pancreas and activation of some components, including resident neutrophils, macrophages, and T cells, which probably play an important role in the impairment of islet survival [6,7,8,9,10].

Some studies have shown that inhibitors of neutrophil elastase (NE), such as sivelestat and α1-antitrypsin, could contribute to the improvement of islet isolation and transplantation [11,12,13,14]. NE is a serine protease stored in azurophilic granules of neutrophils in its inactive form [15,16,17]. When NE is released after neutrophil exposure to inflammatory stimuli, it is fully active. Excessive release of NE degrades several extracellular matrix components, such as elastin, laminins, and collagens, resulting in subsequent tissue damage [15,16,17,18,19]. NE activity reportedly increases during islet isolation, especially at the end of warm digestion by collagenase [11]. Supplementation of preservation solutions with sivelestat, a serine protease inhibitor, significantly improves islet yield and viability. Furthermore, treatment with sivelestat prolongs islet allograft survival in recipient mice [11]. In rats with hyperketonaemia, treatment with sivelestat sodium reduces serum cytokines and improves islet yields and functions in vitro [12]. α1-Antitrypsin is also a serine protease inhibitor that inhibits NE [20,21,22,23], prolongs islet graft survival [13], and reduces expression of proinflammatory mediators [14] in mice by binding to gp96 (a heat-shock protein) [24].

Alvelestat (AZD9668, MPH966) is another NE inhibitor that is reversible and highly selective in biochemical and cellular assays. The inhibitory effect on NE induced by alvelestat is higher than that induced by sivelestat. It has been reported that alvelestat is effective in in vivo mouse and rat models of lung inflammation and degradation [25]. In rats, alvelestat reduces the diameters of abdominal aortic aneurysms (AAA) induced by injection of *Porphyromonas gingivalis* and limits the formation of calcium phosphate precipitation in AAA [26]. In mice with acute lung injury or acute respiratory distress syndrome, alvelestat exhibits potent cytoprotective and anti-inflammatory properties in human alveolar and bronchial epithelial cells by targeting neutrophil extracellular traps, improves survival rates, and reduces lung inflammation [27]. In clinical trials, alvelestat has been shown to reduce sputum inflammatory biomarkers in cystic fibrosis [28] and bronchiectasis [29]. In this study, we investigate whether the addition of alvelestat to preservation solution improves porcine islet yield and function.

## 2. Materials and Methods

### 2.1. Inhibitory Effects of Alvelestat against the Cytotoxicity of NE

Isolated porcine islets from pancreas preservation solution without alvelestat were cultured with Connaught Medical Research Laboratories 1066 medium (CMRL 1066; Sigma-Aldrich Japan, Tokyo, Japan) supplemented with 0.5% human serum albumin (HSA; Sigma-Aldrich Japan) with or without 1 μg/mL NE (Sigma-Aldrich Japan) and 0–25 μM alvelestat (AdooQ Bioscience, Irvine, CA, USA) for 24 h at 37 °C in 5% CO_2_. The islets were evaluated by double fluorescein diacetate/propidium iodide (FDA/PI; Sigma-Aldrich Japan) staining as described previously [30,31,32,33].

### 2.2. Pancreas Preservation Solution

A commercially available extracellular-type trehalose-containing Kyoto (ETK) solution (Otsuka Pharmaceutical Factory, Naruto, Japan) supplemented with 5 μM alvelestat was used as preservation solution. Dimethyl sulfoxide (DMSO; Sigma-Aldrich Japan) plus phosphate-buffered saline (PBS; Sigma-Aldrich Japan) was used to dissolve alvelestat (alelestat(+)), and DMSO plus PBS without alvelestat (alvelestat(−)) was used as the vehicle control.

### 2.3. Procurement, Preservation, and Islet Isolation of Porcine Pancreata

We obtained 20 porcine pancreata (female; three years) from a local slaughterhouse. The operation was started approximately 10 min after the animal’s heartbeat stopped. All pancreata were removed using a standardised technique to minimise warm ischemic time (WIT). We inserted a catheter into the main pancreatic duct and infused preservation solution through the intraductal cannula (1 mL/g of pancreas) [34,35]. The pancreata were then stored in preservation solution at 4 °C for approximately 18 h. The “operation time” was defined as the time from the start of the operation until the removal of the pancreas. The WIT was defined as the time from the cessation of the animal’s heartbeat until placement of the pancreas into the preservation solution. The cold ischemic time, phase I period, and phase II period were defined as described previously [5,35]

To isolate the islets, the ducts were perfused in a controlled fashion with a cold enzyme blend of Liberase T-Flex (1.0 mg/mL) with thermolysin (0.075 mg/mL) (Roche Diagnostics Corporation, Indianapolis, IN, USA). The islets were then separated by gentle mechanical dissociation [5,35,36,37,38,39] and purified using a continuous gradient of purification solution [40]. To generate purification solutions, iodixanol was combined with preservation solution. We adopted bottle purification (size 500 mL; Nalgene, Rochester, NY, USA) in this step [35,40]. The digested tissue was divided in half so that equal amounts of tissue were used for each bottle. A gradient was generated using a gradient marker (Biorep Technologies, Miami Lakes, FL, USA), and candy-cane-shaped stainless steel pipes (length, 30 cm; UMIHIRA, Kyoto, Japan) to enable loading from the low-density solution to high-density solution, leaving the stainless steel pipe in place. After generating a continuous gradient, the digested tissue was loaded as the top layer [40]. The bottles were centrifuged at 1000 rpm (235× *g*) for 5 min at 4 °C. After centrifugation, about 9 fractions (50 mL each) were collected and examined for purity.

### 2.4. Assessment of Islet Function

Dithizone (DTZ; Sigma-Aldrich) staining, scoring of gross morphology (score), and double FDA/PI staining were performed as described previously [1,30,31,32,33,35]. The crude number of islets in each diameter class was determined by counting islets after DTZ staining using an optical graticule. The crude number of islets was then converted to the standard number of islet equivalents (IE; diameter standardized to 150 µm) [1,30,31,32,33,35]. Islet function was assessed by monitoring the insulin secretory response of the purified islets during glucose stimulation using the procedure described previously [1,2,35,41,42,43]. The data were expressed as the mean ± standard error of the mean (SE).

### 2.5. Measurement of ATP Production

To measure the production of ATP, isolated islets in each group were cultured overnight with CMRL-1066 medium supplemented with 0.5%HSA, washed twice with ice-cold PBS, and solubilized. The amount of ATP was measured using an ATP assay system (Toyo Inki, Tokyo, Japan) according to the manufacturer’s instructions. Briefly, after allowing the reagents to equilibrate to room temperature, 10 μL of cell extract was added to 100 μL of reagent. The samples were measured using a luminometer [35].

### 2.6. In Vivo Assessment

For in vivo assessment of the islet function, nude mice (six-week-old, male; Charles River Laboratories Japan, Inc., Kanagawa, Japan) rendered diabetic by a single injection of streptozotocin (STZ) at a dose of 120 mg/kg were used. When the non-fasting blood glucose level exceeded 350 mg/dL for 2 consecutive days, the mice were considered to be diabetic. Marginal numbered porcine islets (1500 IEs) obtained from each group were transplanted into the renal subcapsular space of the left kidney of an immunodeficient diabetic mouse immediately after isolation [33,34,39,41]. Normoglycemia was defined as two consecutive blood glucose level measurements of less than 200 mg/dL. All animal studies were approved by the Institutional Animal Care and Use Committee of the University of the Ryukyus.

### 2.7. Statistical Analysis

All data are expressed as mean ± standard error. Student’s *t*-test was performed to compare two samples from independent groups using Microsoft Excel. The differences in the duration of graft survival between the groups were evaluated using the Kaplan–Meier log-rank test, which was performed using StatView software. Statistical significance was set at *p* < 0.05.

## 3. Results

### 3.1. Inhibitory Effects of Alvelestat against the Cytotoxicity of NE

**Figure 1:**
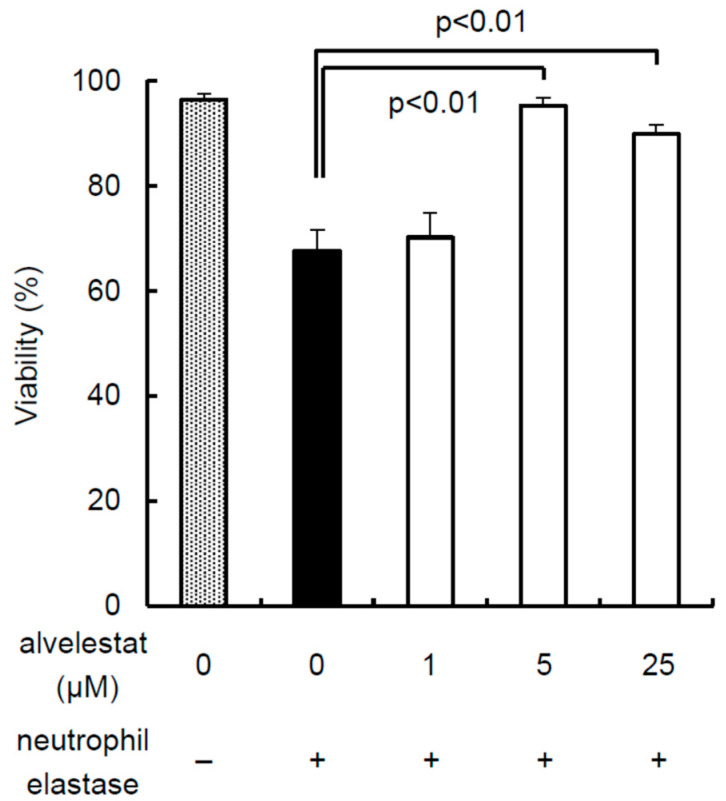
Inhibitory effect of alvelestat against the cytotoxicity of neutrophil elastase. Isolated porcine islets were cultured with or without 1 μg/mL neutrophil elastase and 0–25 μM alvelestat for 24 h. The islets were evaluated using double fluorescein diacetate/propidium iodide (FDA/PI) staining.

To evaluate the inhibitory effect of alvelestat against the cytotoxicity of NE, isolated porcine islets from pancreas preservation solution without alvelestat were cultured with or without 1 μg/mL NE and 0–25 μM alvelestat for 24 h. Islet viability was decreased as a result of treatment with NE and was recovered by treatment with 5 and 25 μM alvelestat but not by treatment with 1 μM alvelestat (Figure 1). The following experiments were performed using 5 μM alvelestat.

### 3.2. Effect of Alvelestat on Porcine Islet Isolation

**Figure 2:**
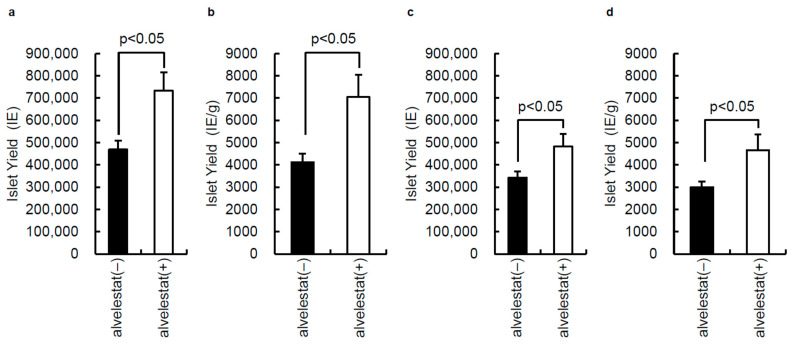
Effect of alvelestat on islet isolation. Porcine pancreata were preserved with or without 5 μM alvelestat for approximately 18 h, and islet isolation was performed. (**a**) Islet yield before purification. (**b**) Islet yield per gram of pancreas before purification. (**c**) Islet yield after purification. (**d**) Islet yield per gram of pancreas after purification. Alvelestat (−) group: *n* = 10; alvelestat (+) group: *n* = 10. Data are expressed as the mean ± SE.

To assess the effect of alvelestat on islet isolation, we performed 20 porcine islet isolations from 21 November 2020 to 6 November 2021. Porcine pancreata were preserved with or without 5 μM alvelestat for approximately 18 h, and islet isolations were performed. The characteristics of porcine pancreata and islets before purification are summarized in Table 1. There were no significant differences in pancreas size, the operation time (the time elapsed between the start of the operation and removal of the pancreas), warm ischemic time (the time elapsed between the removal of the animal’s blood and placement of the pancreas into the preservation solution), cold ischemic time (the time elapsed between the placement of the pancreas into the preservation solution and the start of islet isolation), phase I (digestion time), or phase II (collection time) between the two groups. In contrast, the islet yield before purification of the alvelestat (+) group (*n* = 10) was significantly higher than that of the alvelestat (−) group (*n* = 10) (*p* < 0.05; Figure 2a,b).

The islet characteristics after purification are presented in Table 1. The yield of islets after islet purification of the alvelestat (+) group was significantly higher than that of the alvelestat (−) group (*p* < 0.05; Figure 2c,d). No other characteristics were significantly different between the two groups.

### 3.3. In Vitro Assessment of Isolated Islets from Porcine Pancreata Preserved with or without Alvelestat

**Figure 3:**
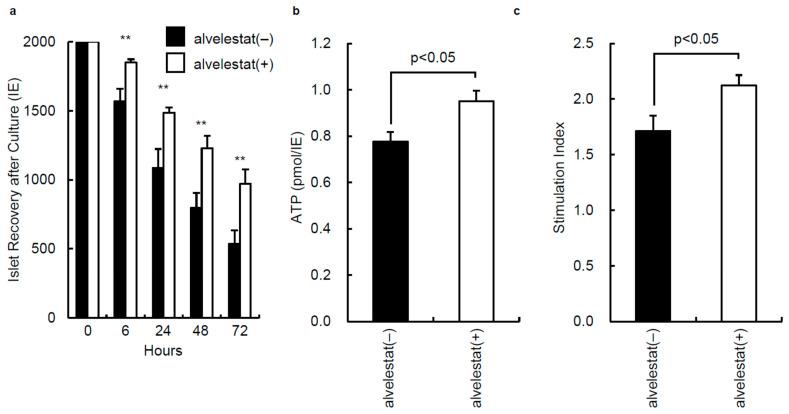
In vitro effect of alvelestat on isolated porcine islet function. (**a**) Number of islets after culturing. Immediately after islet isolation, 2000 IE from each group (*n* = 3 each) were cultured for 72 h. The islets were counted to calculate the IE for each group after 6, 24, 48, and 72 h of culturing. ** *p* < 0.01. (**b**) The adenosine triphosphate (ATP) content in each group was measured using an ATP assay system. Alvelestat (−) group: *n* = 10; alvelestat (+) group: *n* = 10. (**c**) The stimulation index was measured in each group. Alvelestat (−) group: *n* = 10; alvelestat (+) group: *n* = 10. Data are expressed as the mean ± SE.

To investigate whether the addition of alvelestat to preservation solution can prevent the reduction in the number of islets during culturing, the IE was compared between the two groups after culturing for 6, 24, 48, and 72 h. The IE in the alvelestat (−) group (*n* = 3) were significantly lower than the alvelestat (+) group (*n* = 3) (Figure 3a). These data show that the addition of alvelestat to the preservation solution prevents a reduction in the number of islets during culture.

To evaluate islet function in vitro, the ATP content in each group was measured. The ATP content in the alvelestat (−) group (*n* = 10) was significantly lower than that in the alvelestat (+) group (*n* = 10) (Figure 3b). The stimulation index of isolated islets was also measured. The insulin production according to high glucose and stimulation index values in the alvelestat (+) group (*n* = 10) were significantly higher than those in the alvelestat (−) group (*n* = 10) (Figure 3c).

### 3.4. In Vivo Assessment of Porcine Islets from Alvelestat (+) and Alvelestat (−) Groups

To assess the islet graft function in vivo, marginal numbered porcine islets (1500 IEs) from each group (*n* = 20 from 10 islet isolations, *n* = 2 per isolation) were transplanted immediately after isolation under the kidney capsule of STZ-induced diabetic nude mice. The blood glucose levels in 11 of 20 mice (55%) from the alvelestat (+) group decreased and reached normoglycaemia. In contrast, 19 of 20 mice (95%) from the alvelestat (−) group were hyperglycaemic (Table 2). Thus, the number of mice that became normoglycemic after islet transplantation differed significantly between the alvelestat (+) and alvelestat (−) groups (*p* < 0.01). These results revealed that pancreatic preservation with alvelestat improved islet graft function.

## 4. Discussion

In this study, we showed that the addition of alvelestat to the preservation solution increased islet yield and improved the function of isolated islets both in vitro and in vivo. These findings imply that alvelestat has protective effects on the preservation of the pancreas and islets. Donor pancreata are damaged by hypotension, tissue ischemia due to peripheral vasoconstriction, and release of stress hormones and inflammatory cytokines [44,45,46,47]. Donor pancreata are then exposed to warm/cold ischemia during procurement and organ preservation [48,49]. Furthermore, islet isolation, which includes warm digestion, tissue shaking, and cold purification/washing with hypoxia, causes cell damage [44]. It has been reported that NE activity increases during islet isolation, especially at the end of warm digestion by collagenase, with cytotoxic effects against isolated islets [11]. NE injures the membrane of macrophages, acinar cells, and islets [11]. NE also causes the activation of macrophages and acinar cells, leading to proinflammatory cytokines, such as TNF-α and IL-1β [50]. Therefore, alvelestat, sivelestat, and α1-antitrypsin can improve the outcome of islet isolation and transplantation.

ETK solution was used as the preservation solution in this study. We previously reported that a modified ETK solution containing a trypsin inhibitor (ulinastatin) significantly improved the islet yield compared with preservation in the University of Wisconsin (UW) solution for islet isolation [39,51]. ETK solution is advantageous for islet transplantation because the inhibitory effect of collagenase activity by ETK solution is weaker than that of UW solution [39]. Moreover, ETK solution has a high-sodium/low-potassium composition, whereas UW solution has a low-sodium/high-potassium composition, and it is well-known that a high potassium concentration induces insulin release from islets [39]. It has also been reported that the number of neutrophils in the preserved pancreas is significantly reduced by ETK solution with sivelestat compared with UW solution with sivelestat and that the NE activity is significantly suppressed in ETK solution with sivelestat compared with UW solution with sivelestat [11]. Therefore, we chose to use ETK solution and not UW solution. We also reported that ETK solution with ulinastatin is better for pancreas preservation before islet isolation than HTK solution with ulinastatin due to the differences in terms of energy sources of the solutions [52]. Based on these data, we used ETK solution with ulinastatin for clinical islet transplantation from donation after brain death, donation after circulatory death, and living donation [53].

In this study, 5 μM alvelestat was added to the preservation solution based on the data shown in Figure 1. It has been reported that 20 μM sivelestat is a suitable concentration for preservation solutions [11]. The IC50 of sivelestat is 44 nM, and the IC50 of alvelestat is 12 nM [54], suggesting that the inhibitory effect on NE induced by alvelestat is higher than that induced by sivelestat. Moreover, alvelestat is at least 600-fold more selective than other serin proteases [54].

Inhibition of NE leads to effective treatment, as observed in various preclinical studies including skin, bowel, and lung inflammation, as well as ischemia-reperfusion injury relevant to stroke, myocardial infarction, and transplantation [55]. Clinical trials in cardiovascular and lung disease with NE inhibitors, such as sivelestat and alvelestat, are ongoing. It has been reported that there is a trend towards reduction in sputum inflammatory biomarkers with statistically significant changes in interleukin(IL)-6, regulated on activation, normal t-cell expressed and secreted (RANTES) and urinary desmosine in alvelestat-treated patients with cystic fibrosis [28] and that significant changes were observed in forced expiratory volume, slow vital capacity, plasma IL-8, and post-waking sputum IL-6 and RANTES levels in alvelestat-treated patients with bronchiectasis [29]. Alvelestat is currently being explored as a potential treatment for α1-antitrypsin deficiency-related lung disease [56].

## 5. Conclusions

Supplementation of alvelestat in preservation solutions could improve islet isolation and transplantation. Our results imply that alvelestat has cytoprotective and anti-inflammatory effects through the inhibition of NE activity in the pancreas. Because alvelestat has now been used in a clinical trial, further studies, including the verification of safety and efficacy of alvelestat, are expected. Moreover, experiments with the human pancreas are required before clinical islet transplantation can be applied to treat people with type 1 diabetes.

## Figures and Tables

**Table 1:** Characteristics of the pancreatic tissue and isolated islets in the alvelestat (−) group and the alvelestat (+) group.

Characteristic	Alvelestat (−) (*n* = 10)	Alvelestat (+) (*n* = 10)
Pancreas size (g)	120.1 ± 8.9	107.9 ± 5.6
Operation time (min)	4.9 ± 0.4	5.2 ± 0.2
Warm ischemic time (min)	27.1 ± 1.2	27.2 ± 0.8
Cold ischemic time (min)	1116.3 ± 9.4	1087.0 ± 12.7
Phase I period (min)	11.1 ± 0.5	11.6 ± 0.5
Phase II period (min)	38.9 ± 0.8	40.5 ± 0.7
Embedded islets (%)	12.0 ± 1.9	12.0 ± 1.3
Viability (%)	96.7 ± 0.5	96.5 ± 0.6
Purity (%)	64.2 ± 2.4	59.8 ± 3.7
Post-purification recovery (%) ^1^	76.3 ± 3.2	67.4 ± 3.1
Score	9.5 ± 0.2	9.7 ± 0.1

The data are expressed as the mean ± SE. ^1^ Post-purification recovery (%) = IE after purification/IE before purification × 100.

**Table 2:** In vivo functional assay.

	Alvelestat (−)	Alvelestat (+)
No. of Transplanted Mice	20	20
No. of Cured Mice	1	11
%	5	55
*p*-Value vs. alvelestat (−)		<0.01

Marginal numbered porcine islets (1500 IEs) from each group were transplanted immediately after isolation into immunodeficient diabetic mice. Normoglycaemia was defined as <200 mg/dL under non-fasting conditions for more than 2 consecutive days.

## Data Availability

The data that support the findings of this study are available from the corresponding author, H.N., upon reasonable request.
